# CircRNA Profiling Identifies Candidates Associated with Abdominal Fat Deposition in Ducks

**DOI:** 10.3390/biology15161405

**Published:** 2026-08-17

**Authors:** Tingting Zhou, Jiaqi Yao, Jiaming Mao, Chunyan Yang, Shaoxuan Gu, Yong Jiang, Hao Bai, Guobin Chang, Guohong Chen, Zhixiu Wang

**Affiliations:** Key Laboratory of Animal Genetics and Breeding and Molecular Design of Jiangsu Province, Yangzhou University, Yangzhou 225009, China; ztt648715244@163.com (T.Z.); kaltist123@163.com (J.Y.); 13952596198@163.com (J.M.); yangcy202109@163.com (C.Y.); m113198392@163.com (S.G.); jiangyong12126@163.com (Y.J.); bhowen1027@yzu.edu.cn (H.B.); gbchang1975@yzu.edu.cn (G.C.); ghchen@yzu.edu.cn (G.C.)

**Keywords:** Peking duck, abdominal fat deposition, circular RNA, adipocyte differentiation, lipid metabolism, ceRNA network, primary preadipocytes

## Abstract

Excessive abdominal fat deposition reduces the meat yield and feed conversion efficiency of meat ducks. To identify circRNAs associated with this trait, this study performed circRNA sequencing on abdominal fat tissue from fat-type (HF) and lean-type (LF) Peking ducks. Among the 16,513 identified circRNAs, a total of 212 differentially expressed (DE) circRNAs were screened between the HF and LF groups. The host genes of the DEcircRNAs were enriched in processes such as glycerol metabolism, lipid storage, and lipolysis, and were involved in the AMPK, Wnt, insulin, and MAPK signaling pathways. RT-qPCR validated the expression differences of 7 candidate circRNAs and their host genes, of which 3 pairs showed co-variation and 4 pairs showed opposite changes. Based on a predictive ceRNA network comprising 33 circRNAs and 84 mRNAs, 4 candidate circRNAs were selected for validation at the cellular level. Oil Red O staining confirmed progressive lipid accumulation during the differentiation of primary preadipocytes. novel_circ_003710 and novel_circ_010207 were upregulated in both the HF group and differentiated cells, while novel_circ_013157 and novel_circ_011631 were downregulated. Time-course analysis revealed that novel_circ_003710 was consistently upregulated, novel_circ_010207 showed delayed upregulation, novel_circ_013157 was transiently downregulated, and novel_circ_011631 was consistently downregulated. This study identified DE circRNAs associated with abdominal fat deposition and the adipogenic differentiation of primary abdominal fat cells, providing candidate molecules for elucidating the post-transcriptional regulation of abdominal fat deposition in meat ducks.

## Simple Summary

Excessive abdominal fat deposition in meat ducks reduces carcass quality and results in wasted feed energy; however, it remains unclear whether circular RNAs (circRNAs) are involved in regulating this trait. In this study, 14-day-old fat-type (HF) and lean-type (LF) Peking ducks were used as study subjects. Through circRNA sequencing of abdominal adipose tissue, 212 differentially expressed circRNAs were identified. RT-qPCR was used to validate the expression of 7 candidate circRNAs and their host genes; among these, 3 pairs exhibited synergistic changes, while 4 pairs showed opposite changes. By combining ceRNA network predictions with changes observed during the adipogenesis of primary abdominal preadipocytes, 4 circRNAs were further identified: novel_circ_003710 and novel_circ_010207 were consistently upregulated in the HF group and during preadipocyte differentiation, whereas novel_circ_013157 and novel_circ_011631 exhibited a downregulated or initially suppressed, followed by upregulated dynamic pattern. These results indicate that differences in circRNA expression are closely associated with abdominal fat accumulation and the differentiation process of fat cells in ducks. They provide candidate molecular resources for elucidating the post-transcriptional regulatory mechanisms underlying fat deposition in meat ducks, and may serve as a reference for future molecular breeding strategies aimed at reducing abdominal fat and improving carcass quality.

## 1. Introduction

Abdominal fat is a key trait in modern meat duck production that combines both economic importance and biological complexity. Unlike intramuscular fat, which helps improve meat flavor [1], excessive abdominal fat deposition not only runs counter to consumer demand for low-fat, healthy meat products, but also reduces carcass yield and feed conversion efficiency, while increasing resource wastage during slaughter and processing [2,3]. Therefore, while maintaining stable growth performance and meat quality, elucidating the genetic and molecular mechanisms that specifically regulate abdominal fat deposition has become a critical scientific issue for precision breeding and fat trait improvement in meat ducks.

Lipid metabolism in poultry exhibits distinct species-specific characteristics. Unlike in mammals, fatty acid synthesis in poultry occurs primarily in the liver, while abdominal adipose tissue is responsible for functions such as lipid uptake, storage, mobilization, and tissue remodeling [4]. In poultry, abdominal adipose tissue grows significantly faster than other types of adipose tissue and therefore plays a particularly important physiological role [5]. However, abdominal fat deposition is not linearly determined by a single coding gene; rather, it is a complex quantitative trait shaped jointly by mRNAs, non-coding RNAs (ncRNAs), and their interactive networks. Analyzing this process solely at the mRNA level makes it difficult to fully elucidate its regulatory mechanisms.

Circular RNA (circRNA) is a covalently closed, non-coding RNA formed by reverse splicing. Due to the absence of a 5′ cap and a poly(A) tail, it is highly stable and exhibits tissue-, cell type-, and developmental stage-specific expression [6,7]. Functionally, circRNAs can act as a miRNA sponge, regulate host gene transcription and splicing, bind to RNA-binding proteins [8,9], or be translated into peptides or proteins under specific conditions [10]. These characteristics suggest that their formation may be finely regulated by the cell’s differentiation state.

Previous transcriptomic studies of HF and LF Peking ducks revealed adipose depot-specific differences in gene expression but did not examine circRNAs [11]. In duck adipose models, circRNA profiling identified differentiation-associated circRNAs in subcutaneous preadipocytes and demonstrated a role for circ-PLXNA1 in adipogenesis [12]. Differentially expressed circRNAs associated with PPAR signaling and triglyceride metabolism have also been identified in abdominal adipose tissue from ducks with high and low abdominal fat percentages [13], while more recent work has suggested depot-specific circRNA–miRNA networks during intramuscular and subcutaneous adipocyte differentiation [14]. However, these studies examined in vivo adipose phenotypes and in vitro adipocyte differentiation separately. It therefore remains unclear which circRNAs associated with divergent abdominal fat deposition in Peking ducks also exhibit dynamic expression during abdominal preadipocyte differentiation.

This study focused on 14-day-old fat-type (HF) and lean-type (LF) Peking ducks, conducting circRNA sequencing on abdominal adipose tissue to systematically screen for candidate circRNAs associated with lipid metabolism and adipogenesis. RT-qPCR was then used to detect differences in the expression of candidate circRNAs and their host genes in the abdominal adipose tissue of HF and LF groups of Peking ducks. Furthermore, an independent duck primary abdominal preadipocyte model was utilized, combined with simultaneous uninduced controls and continuous differentiation time points, to analyze the expression dynamics of key candidate circRNAs during adipogenesis. The aim of this study is to screen for circRNAs that are simultaneously associated with the abdominal fat phenotype of Peking ducks and duck preadipocyte differentiation, prioritizing circRNAs related to both abdominal fat divergence and abdominal adipogenesis, and to provide focused candidates for subsequent mechanistic validation.

## 2. Materials and Methods

### 2.1. Ethical Approval

Experimental procedures were performed by professionally trained personnel. While ensuring the effectiveness of the procedures, the duration of each procedure was minimized, and gentle restraint methods were employed to reduce stress on the animals. Prior to the start of the experiment, the ducks underwent an acclimatization period to alleviate stress caused by the environmental change. All animal handling strictly adhered to relevant regulations regarding the welfare of laboratory animals. All experimental procedures, including the handling of animals, were conducted in strict accordance with the experimental protocol approved by the Yangzhou University Committee on Animal Care and Use (Protocol No. SYDW-2019015).

### 2.2. Animals and Sample Preparation

The meat ducks used in this study for sequencing were 14-day-old fat-type (HF, *n* = 4) and lean-type (LF, *n* = 4) Peking ducks (Cangzhou, Hebei, China), randomly selected from each group without sex distinction. The HF and LF Peking ducks represent two distinct genetic lines with divergent fat deposition traits, which were developed through multiple generations of selective breeding in opposite directions. The LF line is characterized by a high lean meat percentage and low abdominal fat, while the HF line is characterized by a high body fat content and rapid subcutaneous and abdominal fat deposition. For the first 14 days after hatching, the ducks were reared contemporaneously under the same feeding and management conditions, including the same diet, housing system, stocking density, lighting schedule, ambient temperature, and access to feed and water, and their health condition was good. Live body weight was recorded after a 12 h fast prior to slaughter. Immediately after euthanasia, abdominal fat weight was measured by collecting the fat surrounding the gizzard, proventriculus, and the abdominal cavity in each duck on a clean operating table and weighing it. Abdominal fat percentage (AFP) was determined relative to live body weight using the following formula: $AFP\;\left(\%\right)=\frac{Abdominal\;Fat\;Weight\left(g\right)}{Live\;Body\;Weight\left(g\right)}\times 100\%$. The collected tissue was immediately immersed in liquid nitrogen to freeze it and then stored at −80 °C for future use.

### 2.3. Quality Control of RNA Sequencing Data

To ensure high-quality, clean reads for downstream analysis, a rigorous quality control process was applied to the raw sequencing reads from abdominal fat tissue of fat-type and lean-type Peking ducks. First, Fastp [15] was used to perform rigorous quality control on the raw sequencing data (raw reads) from the abdominal fat tissue of meat ducks. Reads containing adapters, low-quality bases (where bases with a quality score of Q ≤ 20 account for more than 50% of the entire read), and those with a N proportion greater than 10% were removed. FastQC (version 0.12.1; Babraham Bioinformatics, Cambridge, UK; https://www.bioinformatics.babraham.ac.uk/projects/fastqc/, accessed on 14 May 2026) was used to evaluate the base composition and quality distribution of the cleaned data (clean reads) to ensure the accuracy and uniformity of the sequencing.

### 2.4. Read Alignment Analysis

To eliminate ribosomal contamination, the clean reads were aligned to a ribosomal RNA (rRNA) database using BOWTIE2 (Version 2.2.9) [16], and reads that mapped to ribosomes were removed. Next, the unmapped reads were aligned to the meat duck reference genome using HISAT2 (Version 2.2.0) [17], with default parameters. Finally, the distribution of the mapped reads across the genome, including exons, introns, and intergenic regions, was quantified and analyzed to evaluate the mapping characteristics of the transcriptomic data.

### 2.5. Identification and Bioinformatics Analysis of circRNAs

Candidate circRNAs were identified in the abdominal fat of HF and LF ducks using the mapped reads. Their size distribution was established from full-length sequences, and expression abundance was quantified via back-splicing junction (BSJ) reads using CIRIquant [18]. Additionally, circRNAs were annotated and classified based on the reference genome coordinates, and their genomic distribution across autosomes and sex chromosomes was statistically summarized.

### 2.6. Differential Expression and Hierarchical Clustering Analysis of Circular RNAs

Raw read count data obtained from circRNA expression analysis were used as input for differential expression analysis, which was performed using the edgeR package. The analysis workflow primarily included read count normalization, followed by the calculation of *p*-value via hypothesis testing based on generalized linear models in edgeR (Version 3.21) [19]. By setting thresholds of *p*-value < 0.05 and log_2_ fold change (|log_2_ FC| > 1), differentially expressed circRNAs (DE circRNAs) between the HF group and the LF group were identified.

### 2.7. Functional Enrichment Analysis of Host Genes Associated with DE circRNAs

To elucidate the potential biological roles and functional themes of these altered circRNAs, Gene Ontology (GO, http://www.geneontology.org, accessed on 20 June 2026) annotation and Kyoto Encyclopedia of Genes and Genomes (KEGG, http://www.genome.jp/kegg, accessed on 20 June 2026) pathway enrichment analyses were performed on the linear host genes corresponding to the identified differentially expressed circRNAs (DE circRNAs). Terms and pathways with *p*-value < 0.05 are considered as nominatively enriched. The overall enrichment pattern of the top 20 terms and pathways is visualized using a hierarchical pie chart, while specific lipid metabolism-related functional categories and cascades are further illustrated using a directed bubble chart.

### 2.8. Construction of a ceRNA Regulatory Sankey Diagram

To elucidate the multilevel regulatory flows and co-expression relationships between key circRNAs and their lipid-forming targets, a comprehensive Sankey diagram was constructed. Pearson correlation analysis was performed on the expression matrices of DE circRNAs and ceRNA-predicted target transcripts across all biological replicate samples. Pairs with high synchrony meeting the thresholds of r > 0.90 and *p*-value < 0.05 were selected. The filtered interlocking relationships among candidate circRNAs, functional GO terms, and KEGG pathway-associated target genes were systematically mapped and visualized via a Sankey framework to trace the directional regulatory streams driving abdominal fat deposition.

### 2.9. Cell Culture

Abdominal fat and abdominal preadipocytes were isolated from 14-day-old Cherry Valley ducks (Suqian, China) via digestion with type I collagenase (Sigma 1 g, Saint Louis, MO, USA) and the differential adhesion method. Isobutylmethylxanthine (IBMX, 10 mg, Sigma, St. Louis, MO, USA), insulin (10 mg/mL, Cat. No. PB180432, Procell Life Science and Technology Co., Ltd., Wuhan, China), rosiglitazone (RSG 100 mg, MedChemExpress, Monmouth Junction, NJ, USA), and dexamethasone (Sigma 1 mM, St. Louis, MO, USA) were used to induce preadipocytes. Primary abdominal preadipocytes cultured normally without induction were designated as ABF-Z2, ABF-Z4, and ABF-Z6, respectively. Primary abdominal preadipocytes were induced using the induction systems for 2, 4, and 6 days and were designated as ABF-Y2, ABF-Y4, and ABF-Y6, respectively. All culture systems were maintained at 37 °C, 95% humidity, and 5% CO_2_ (Thermo Fisher Scientific, Waltham, MA, USA). Primary abdominal preadipocytes isolated from the same pool of adipose tissue were seeded into multiple independent culture wells under identical conditions. For temporal expression analysis, cells from five independent wells were harvested at each indicated time point (Days 0, 2, 4, 6, and 8) during the adipogenic differentiation process. Thus, each time point consisted of five independent biological replicates derived from separate wells.

### 2.10. Total RNA Extraction, cDNA Synthesis, and Quantitative Real-Time PCR (qRT-PCR)

Total RNA was extracted from tissue and cell samples, respectively. cDNA was synthesized using the *Evo M-MLV* Reverse Transcription Premix Kit Ver. 2 (AG11728, Accurate Biotechnology Co., Ltd., Changsha, China). The primers used for qRT-PCR were designed using primer3plus (https://primer3.ut.ee/, accessed on 22 June 2026) (Table S1). To specifically detect and quantify circRNAs and distinguish them from their linear counterparts, divergent primers were designed to span the backsplice junction of each target circRNA. The BSJ sequence for each circRNA was determined based on our RNA sequencing data. Unlike conventional convergent primers, this divergent primer pair faces away from each other on the linear RNA template. Consequently, the primers can only amplify the circularized transcript across the BSJ and cannot amplify the linear mRNA from the same host gene. qRT-PCR experiments were performed using the SYBR qPCR Master Mix Kit (Vazyme, Shanghai, China) on the FQD-96A detection system (Bioer Technology, Hangzhou, China). GAPDH and β-actin were used as internal controls for circRNA and mRNA, respectively. Gene expression levels were calculated using the 2^−ΔΔCt^ method.

### 2.11. Statistical Analysis

All statistical analyses were performed using SPSS 22.0 (SPSS Inc., Chicago, IL, USA) and GraphPad Prism 10.0 (GraphPad Software, San Diego, CA, USA). Data are expressed as mean ± standard error of the mean (S.E.M.). For two-group comparisons, statistical significance was evaluated using two-tailed Student’s *t*-tests. For temporal expression dynamics during adipogenic differentiation involving multiple time points, differences relative to the baseline control (Day 0) were analyzed using one-way analysis of variance (ANOVA) followed by Dunnett’s post hoc test. * *p*-value < 0.05 and ** *p*-value < 0.01 were considered statistically significant and highly statistically significant, respectively. Bar graphs illustrating the results were generated using GraphPad Prism 10 software.

## 3. Results

### 3.1. Abdominal Fat Phenotypes of HF and LF Peking Ducks

As shown in Table 1, the abdominal fat mass and abdominal fat percentage in the HF group were significantly higher than those in the LF group (*p*-value < 0.01). The complete phenotypic metrics for all animals in each group of qRT-PCR validation are comprehensively provided in Supplementary Table S2.

### 3.2. Quality Assessment and Reference Genome Alignment of circRNA Sequencing Data from HF and LF Peking Ducks

**Figure 1:**
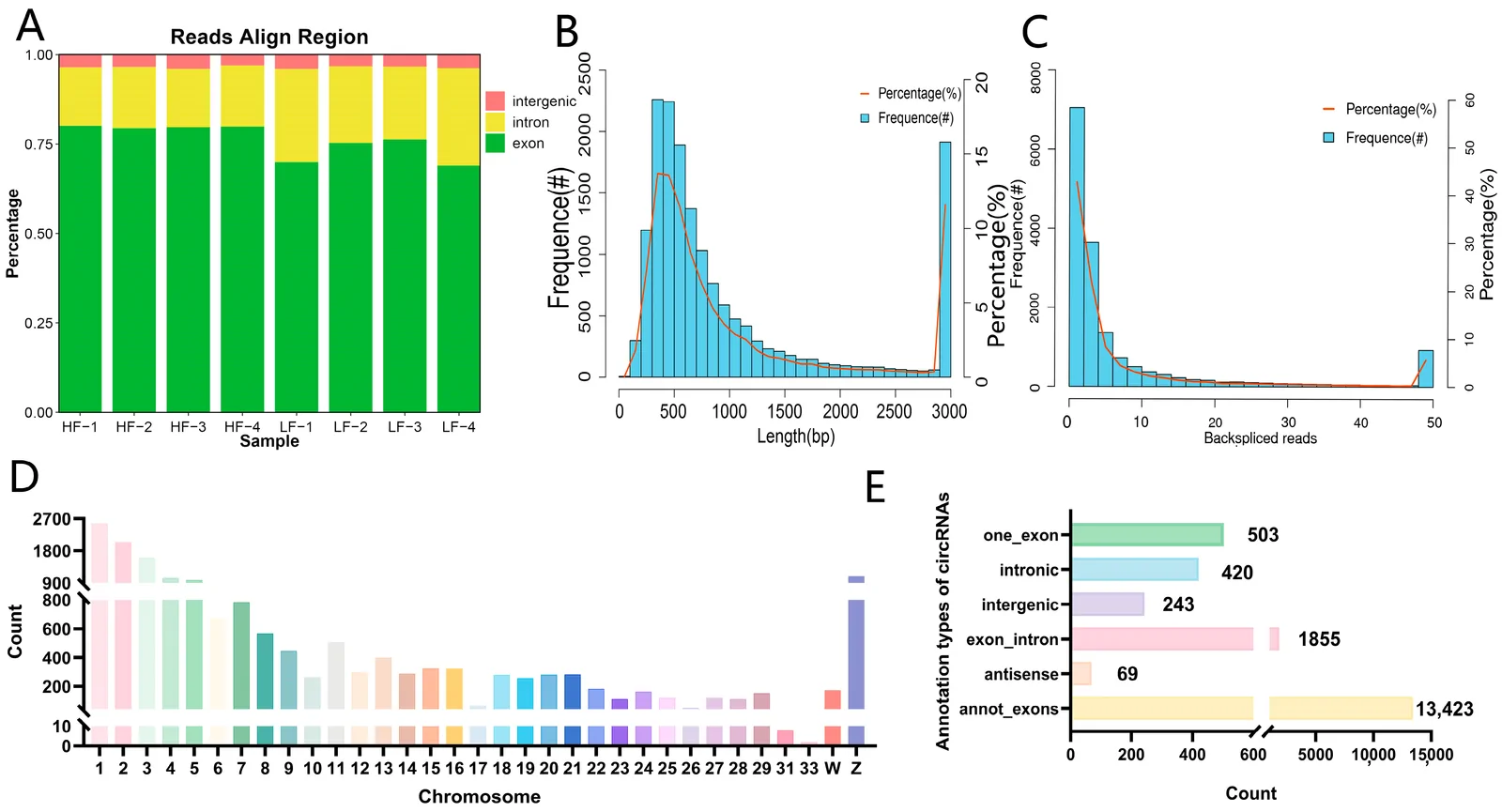
Alignment characteristics and genomic features of circRNA sequencing reads from abdominal adipose tissue of Peking ducks. (**A**) Distribution of mapped reads in exons, introns, and intergenic regions in the HF and LF groups. (**B**) Histogram of the length distribution of identified circRNAs. (**C**) Distribution of circRNA expression abundance based on the number of reads supported by back-splicing junctions (BSJ). (**D**) Chromosomal distribution map of identified circRNAs. (**E**) Distribution map of circRNA types.

To analyze the circRNA expression patterns associated with abdominal fat deposition in Peking ducks, this study collected abdominal fat tissue from 14-day-old Peking ducks (HF) and lean-type (LF) Peking ducks. After removing ribosomal RNA (rRNA) and treating the samples with RNase R, circRNA sequencing libraries were constructed. A total of 548,043,298 raw sequencing reads were obtained from the 8 samples. After adapter removal and low-quality read filtering, the average retention rates of the raw data for the HF and LF groups were 99.46% and 99.56%, respectively (Table S3). The base composition of the sequencing data across all samples was generally stable, with an average Q30 percentage of 92.74% (Table S4), indicating that the sequencing data was of good quality and suitable for subsequent analysis. rRNA alignment results showed that only a small number of sequencing reads in each sample aligned to rRNA sequences (Table S5), indicating that rRNA removal was effective. Further alignment of the filtered high-quality reads to the duck reference genome (NCBI_GCF_015476345.1) revealed that the average unique alignment rates for the HF and LF groups were 81.79% and 80.75%, respectively (Table S6). Analysis of genomic region distribution showed that the sequencing reads from both groups were primarily localized to exonic regions, accounting for an average of 76.22%; followed by intron regions and intergenic regions, accounting for 20.24% and 3.55%, respectively (Figure 1A and Table S7). Overall, the HF and LF groups exhibited high consistency in terms of data filtering rates, reference genome alignment rates, and genomic region distribution, indicating that the sequencing data are of high quality and comparable, providing a reliable data foundation for subsequent circRNA identification and differential expression analysis.

### 3.3. Genomic Characteristics and Expression Abundance of circRNAs in Peking Duck Abdominal Adipose Tissue

To characterize the circular RNAs identified in abdominal adipose tissue from Peking ducks, we analyzed their length distribution, expression abundance, genomic origin, and chromosomal distribution. The identified circular RNAs varied significantly in length, with a peak range between 300 and 500 bp. Beyond this range, the number of circRNAs generally decreased as sequence length increased (Figure 1B). CircRNAs with lengths ≥ 3000 bp constituted an additional fraction of the identified circRNAs. We then estimated the expression abundance of each circular RNA based on the number of reads spanning the back-splicing junction (BSJ). The distribution of BSJ read counts was markedly right-skewed, indicating that the detection abundance of most circular RNAs was relatively low, while only a small fraction exhibited high abundance (Figure 1C). Circular RNAs were distributed across all analyzed chromosomes, with chromosome 1 (Chr1) containing the highest number of identified circular RNAs, followed by chromosome 2 (Chr2), while chromosome 33 (Chr33) contained the fewest (Figure 1D). Based on their genomic positions relative to annotated genes, the identified circular RNAs were classified into six categories: annot_exons, antisense, exon_intron, intergenic, intronic, and one_exon (Figure 1E). Among these categories, annot_exons circular RNAs had the highest abundance and thus constituted the major category of genome-derived circular RNAs in Peking duck abdominal fat tissue.

### 3.4. Differential Expression and Cluster Analysis of circRNAs Between the HF and LF Groups

**Figure 2:**
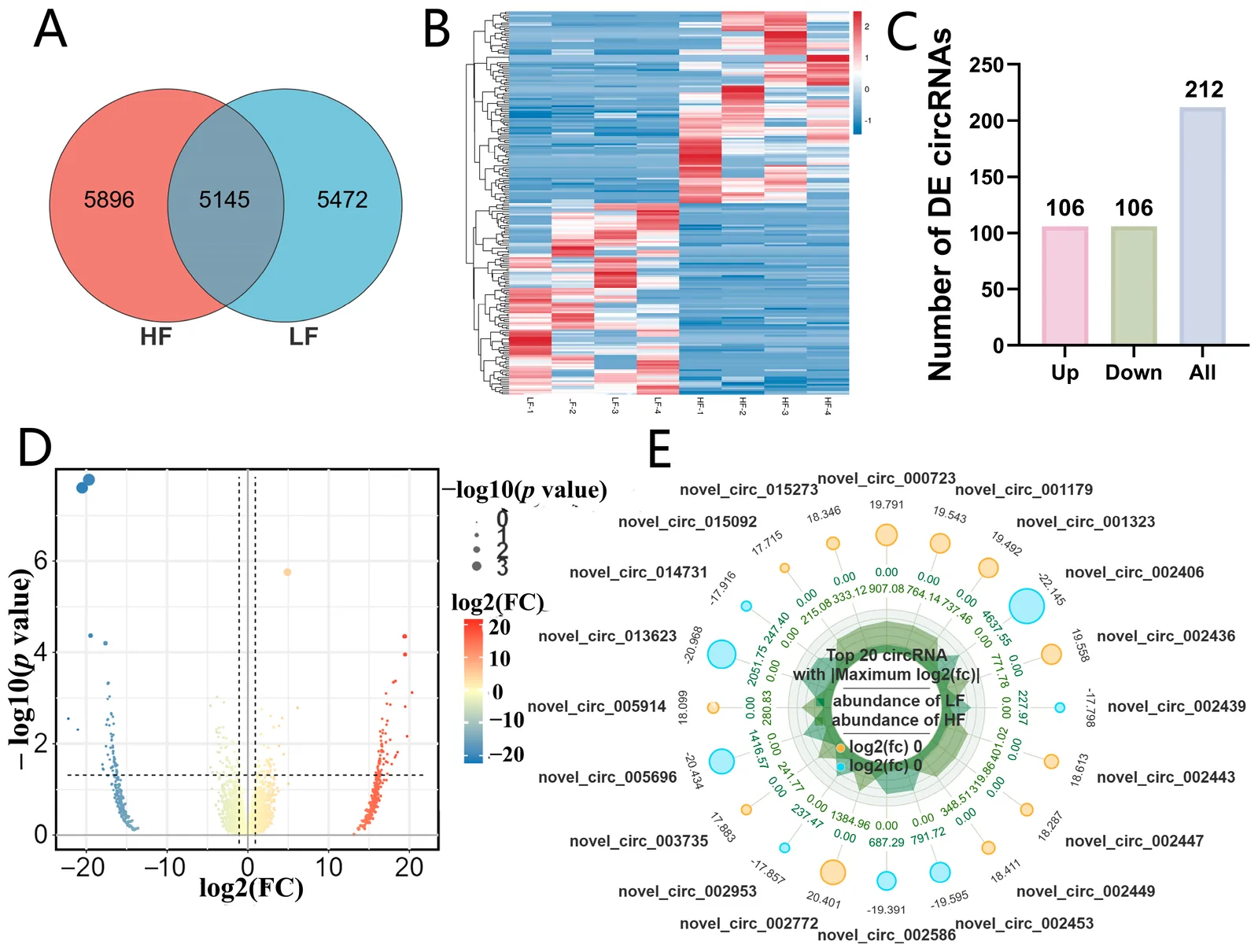
Differential expression profiles and clustering analysis of circRNAs between the HF and LF groups. (**A**) A Venn diagram showing unique and overlapping circRNA candidates identified in abdominal adipose tissue from the HF and LF groups. (**B**) A hierarchical clustering heatmap used to assess the overall expression patterns of 212 significantly DE circRNAs across biological replicates. (**C**) A statistical bar chart summarizing the total number of significantly upregulated and downregulated DE circRNAs. (**D**) A volcano plot showing the overall transcriptional variation and statistical significance of all identified transcripts. Data points are displayed using a unique blue-yellow-red gradient, corresponding to significantly downregulated (blue), statistically insignificant (yellow), and significantly upregulated (red) circRNAs, respectively. (**E**) A radar chart illustrating the multidimensional characteristics of the top 20 differentially expressed circRNAs. These candidate circRNAs were selected and ranked based on the thresholds of *p*-value < 0.05 and |log_2_ FC| ≥ 1, where the orange regions represent significantly upregulated transcripts and the blue regions represent significantly downregulated transcripts. The size of the circle varies depending on the value of log_2_ FC.

A total of 16,513 unique circRNAs were identified in all abdominal fat tissue samples from Peking ducks. Among these, 11,041 and 10,617 circRNAs were detected in the HF and LF groups, respectively, with 5145 circRNAs shared between the two groups. Accordingly, only 5896 and 5472 group-specific circRNAs were detected in the HF and LF groups, respectively (Figure 2A). To identify circRNAs potentially associated with differences in abdominal fat deposition, differential expression analysis was performed between the HF and LF Groups, resulting in the identification of 212 differentially expressed circRNAs (DE circRNAs) (Table S8). Compared with the LF group, 106 DEcircRNAs were upregulated, and 106 were downregulated in the HF group (Figure 2C). Based on the normalized expression levels of these differentially expressed circRNAs (DEcircRNAs), hierarchical clustering analysis was performed to visualize their expression patterns across the eight samples (Figure 2B). In addition, a volcano plot was generated to summarize the magnitude and statistical significance of expression differences between the two groups (Figure 2D). A radar chart was used to further visualize the 20 differentially expressed circular RNAs (DEcircRNAs) with the largest absolute fold changes (Figure 2E). Based on the functional annotations of their host genes, novel_circ_002436, novel_circ_002439, novel_circ_002443, novel_circ_002447, novel_circ_002449, novel_circ_005696, novel_circ_013623, and novel_circ_002453 were prioritized as candidates potentially associated with lipid metabolism and adipogenesis.

### 3.5. GO Functional Annotation and KEGG Pathway Enrichment Analysis of DE circRNAs Host Genes

**Figure 3:**
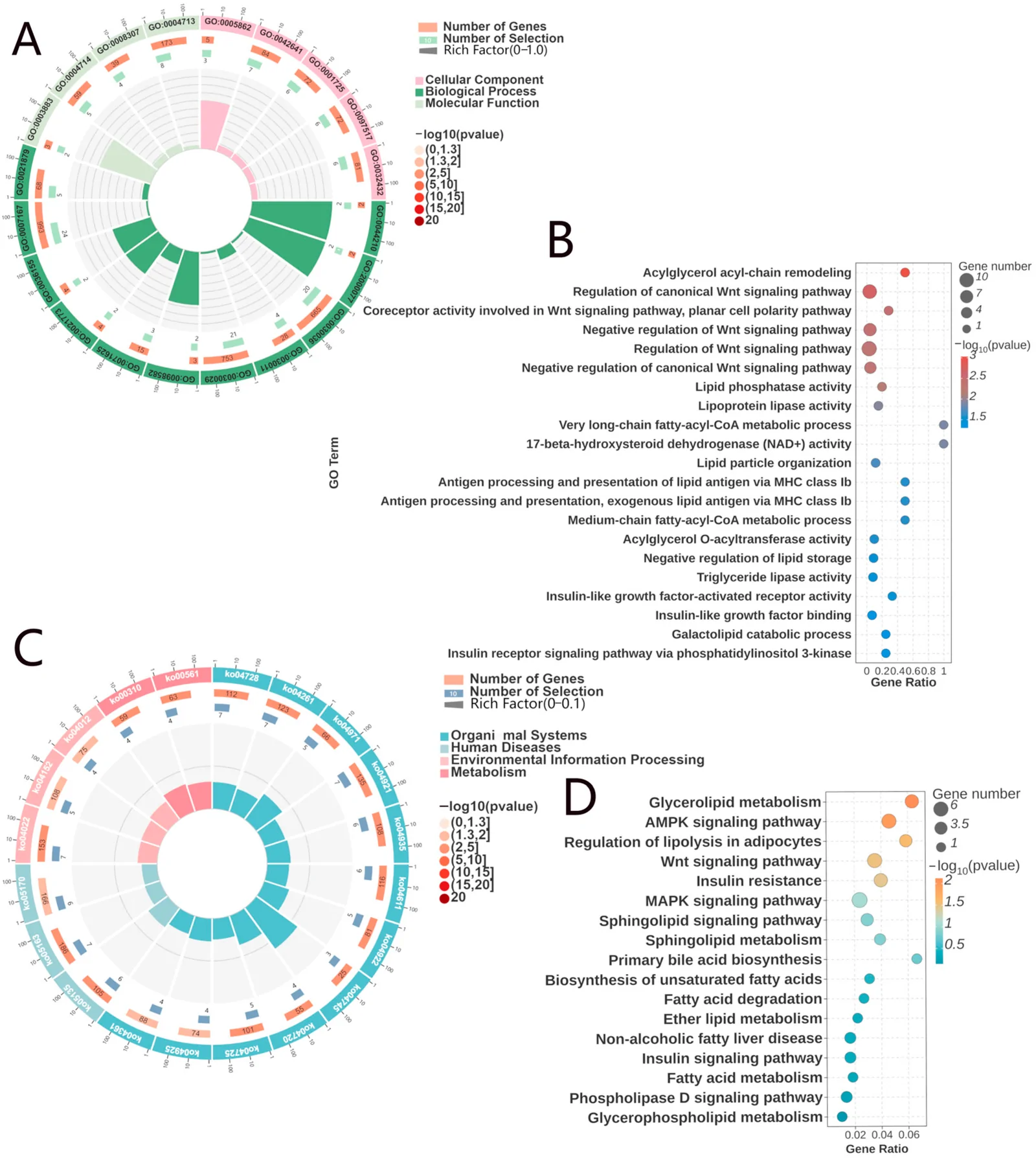
Functional GO annotation and KEGG pathway enrichment analysis of host genes of differentially expressed circular RNAs. (**A**) GO enrichment pie chart showing the 20 most enriched terms across the three major categories: biological processes, cellular components, and molecular functions. The concentric circles, from the outer to the inner, represent, respectively, the coordinate scale for gene counts (color-coded by category), background gene counts and statistical significance (*p*-value), counts of specific differentially expressed circRNA host genes, and Rich factor values plotted against a background grid (0.1 per square). (**B**) A bubble plot showing selected lipid-related GO terms enriched in DE circRNA host genes. (**C**) KEGG enrichment pie chart showing the top 20 enriched pathways. (**D**) A bubble plot showing selected lipid metabolism pathways enriched by differentially expressed circRNA host genes.

To investigate the potential biological functions associated with the DE circRNAs, GO and KEGG enrichment analyses were performed on their host genes. GO annotation assigned these genes to the three major GO categories: biological process, cellular component, and molecular function (Figure 3A). Further examination of lipid deposition-related terms revealed enrichment in biological processes associated with acylglycerol acyl-chain remodeling, very-long-chain and medium-chain fatty acyl-CoA metabolism, lipid particle organization, and the negative regulation of lipid storage. Several lipid metabolism-related molecular functions were also represented, including lipoprotein lipase activity, triglyceride lipase activity, lipid phosphatase activity, acylglycerol O-acyltransferase activity, and 17β-hydroxysteroid dehydrogenase (NAD^+^) activity (Figure 3B). In addition to lipid metabolic functions, multiple terms related to Wnt signaling were identified, including the regulation and negative regulation of both canonical and non-canonical Wnt signaling pathways. Terms associated with insulin-like growth factor binding, insulin-like growth factor-activated receptor activity, and insulin receptor signaling through phosphatidylinositol 3-kinase were also represented. These GO results indicated that the host genes of the DEcircRNAs were functionally associated with lipid metabolism, lipid storage, and signaling processes relevant to adipocyte development and metabolism. KEGG analysis classified the host genes into four major functional categories (Figure 3C). Among the lipid deposition-related pathways examined, glycerolipid metabolism exhibited one of the highest gene ratios and strongest enrichment signals (Figure 3D). The host genes were also enriched or represented in the AMPK signaling pathway, regulation of lipolysis in adipocytes, Wnt signaling pathway, insulin resistance, MAPK signaling pathway, and insulin signaling pathway. In addition, several pathways directly involved in lipid synthesis, degradation, and transport were identified, such as sphingolipid metabolism and signal transduction, biosynthesis of unsaturated fatty acids, fatty acid degradation, and fatty acid metabolism.

Collectively, the GO and KEGG results suggest that the host genes of the DE circRNAs are primarily involved in lipid metabolic processes and adipogenesis-related signaling pathways. These findings provide functional clues that the corresponding DE circRNAs may be associated with differences in abdominal fat deposition between the HF and LF groups; however, their specific regulatory functions require further experimental validation.

### 3.6. Expression Validation of DEcircRNAs and Their Host Genes

**Figure 4:**
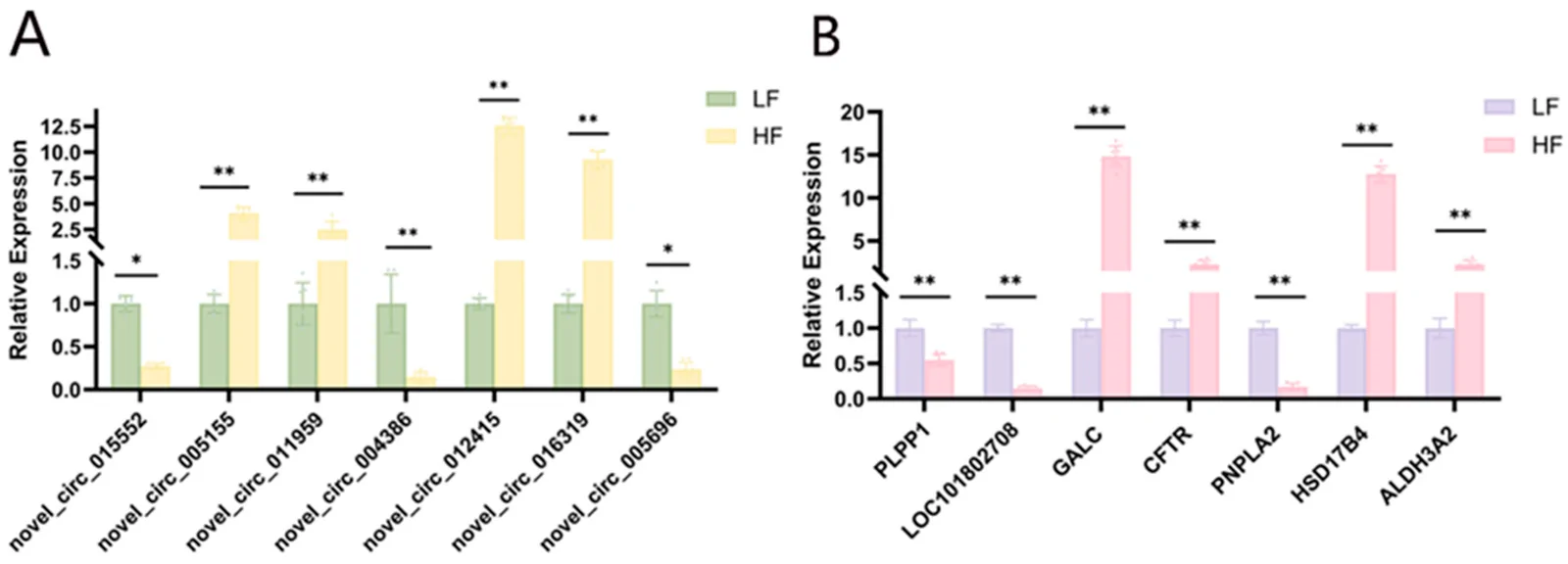
RT-qPCR validation of selected DEcircRNAs and their corresponding host genes in Peking duck abdominal adipose tissue (*n* = 8 per group). (**A**) Relative expression levels of seven selected DEcircRNAs in the HF and LF groups. (**B**) Relative expression levels of host genes corresponding to 7 selected DEcircRNAs. The mean expression level in the LF group was normalized to 1. * *p*-value < 0.05, ** *p*-value < 0.01.

To validate the expression patterns of candidate DE circRNAs associated with lipid metabolism and abdominal fat deposition, we used RT-qPCR to detect the expression of seven DE circRNAs and their corresponding host genes in abdominal adipose tissue from the HF and LF groups (Table 2). The results showed that all 7 candidate DEcircRNAs exhibited significant expression differences between the HF group and the LF group (Figure 4A). Compared with the LF group, novel_circ_005155, novel_circ_011959, novel_circ_012415, and novel_circ_016319 were significantly upregulated in the HF group (*p*-value < 0.01); novel_circ_015552, novel_circ_004386, and novel_circ_005696 were significantly downregulated in the HF group (*p*-value < 0.05). Among the upregulated candidate circRNAs, novel_circ_012415 and novel_circ_016319 exhibited the greatest relative expression differences between the HF and LF groups. The expression levels of the corresponding host genes also differed significantly between the two groups (Figure 4B). Compared with the LF group, *GALC*, *CFTR*, *HSD17B4*, and *ALDH3A2* were significantly upregulated in the HF group, while *PLPP1*, *LOC101802708*, and *PNPLA2* were significantly downregulated in the HF group (*p* < 0.01).

Further comparison of the expression directions of circRNAs and their host genes revealed that both positive and negative expression relationships coexist among different circRNA/host gene pairs. novel_circ_015552/*PLPP1*, novel_circ_011959/*GALC*, and novel_circ_016319/*HSD17B4* exhibited co-directional changes in expression. Specifically, novel_circ_015552/*PLPP1* was significantly downregulated in the HF group, whereas novel_circ_011959/*GALC* and novel_circ_016319/*HSD17B4* were both significantly upregulated in the HF group. In contrast, novel_circ_005155/*LOC101802708*, novel_circ_004386/*CFTR*, novel_circ_012415/*PNPLA2*, and novel_circ_005696/*ALDH3A2* exhibited opposite expression changes. These results validate the differential expression of the candidate circRNAs between the HF and LF groups and indicate that changes in circRNA expression do not always align with the expression trends of their host gene linear transcripts.

**Table 2:** Representative DE circRNAs and their host genes selected for expression validation.

circRNA	Source Gene	Symbol
novel_circ_004386	ncbi_101803386	*CFTR*
novel_circ_005155	ncbi_101802708	*LOC101802708*
novel_circ_005696	ncbi_113839578	*ALDH3A2*
novel_circ_011959	ncbi_101796301	*GALC*
novel_circ_012415	ncbi_101796497	*PNPLA2*
novel_circ_015552	ncbi_101799557	*PLPP1*
novel_circ_016319	ncbi_101791118	*HSD17B4*

### 3.7. Construction of a Putative Lipid Metabolism-Related ceRNA Network and Expression Validation of Candidate circRNAs

**Figure 5:**
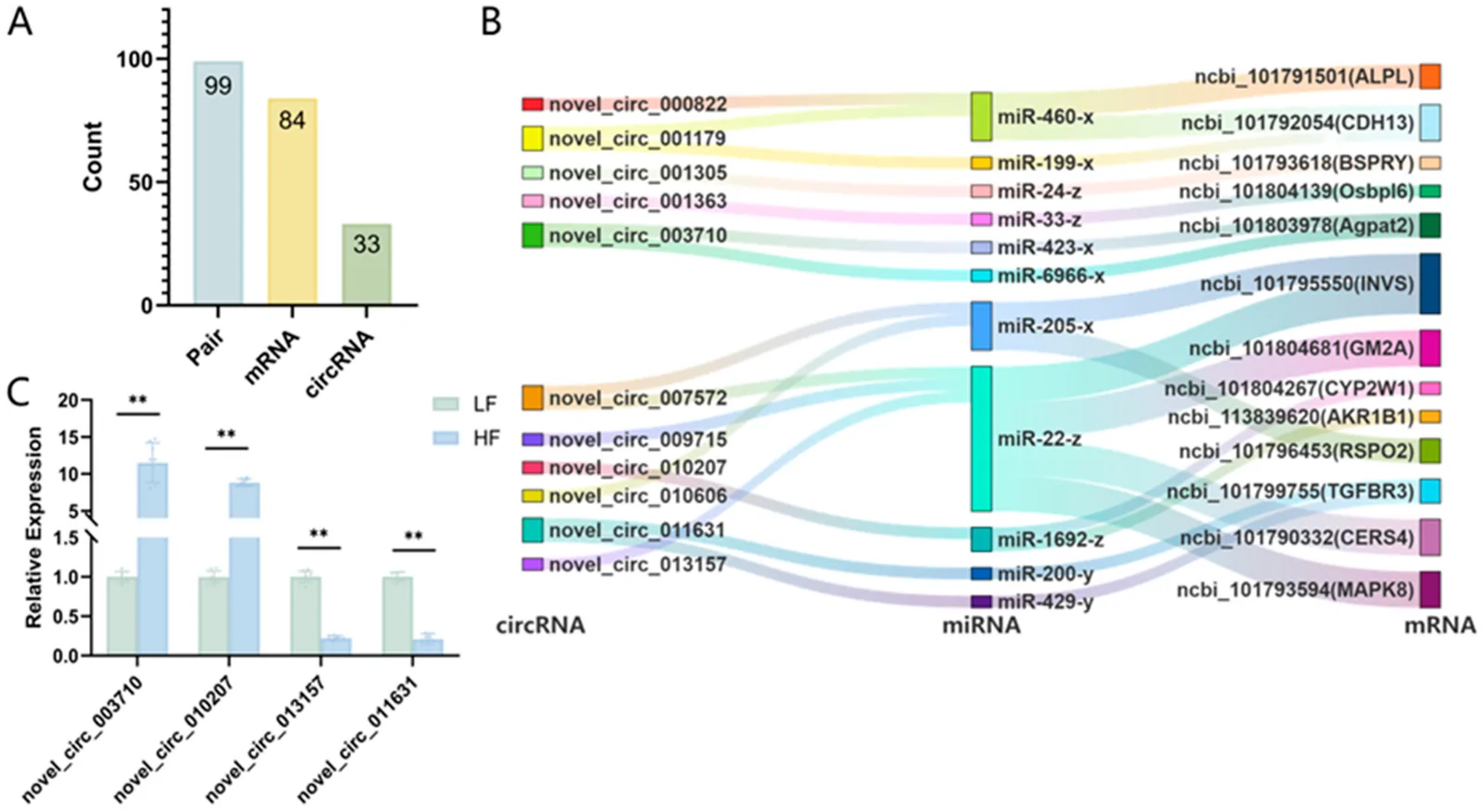
Mapping the ceRNA regulatory network between key circular RNAs and adipogenesis targets. (**A**) Statistical bar chart of the ceRNA network; (**B**) A Sankey diagram illustrating the directed flow and multi-database intersections among candidate circular RNAs (left nodes), miRNAs (middle nodes), and related target genes (right nodes) during abdominal fat metabolism; (**C**) Relative expression levels of candidate circRNAs in abdominal fat tissue from LF group and HF group (*n* = 8 per group, Mean ± S.E.M). ** *p*-value < 0.01.

To identify potential circRNA-mediated regulatory relationships associated with abdominal fat deposition, a putative circRNA–miRNA–mRNA competing endogenous RNA (ceRNA) network was constructed by integrating the predicted circRNA–miRNA and miRNA–mRNA interactions (Table S9). A total of 99 putative circRNA–mRNA regulatory pairs involving 33 circRNAs and 84 mRNAs were identified (Figure 5A). Focusing on genes associated with lipid metabolism, adipogenesis, and related signaling pathways, a representative ceRNA subnetwork comprising 11 circRNAs, 11 miRNAs, and 13 mRNAs was subsequently constructed (Figure 5B). Four circRNAs with prominent positions in the network were selected for RT-qPCR analysis in abdominal adipose tissue from the HF and LF groups. Compared with the LF group, novel_circ_003710 and novel_circ_010207 were significantly upregulated in the HF group, whereas novel_circ_013157 and novel_circ_011631 were significantly downregulated (*p* < 0.01; Figure 5C). These results confirmed the differential expression of the four candidate circRNAs between the HF and LF groups and prioritized them for subsequent investigation of their potential roles in abdominal fat deposition.

### 3.8. Expression Patterns of Candidate circRNAs During the Adipogenic Differentiation of Primary Duck Abdominal Preadipocytes

**Figure 6:**
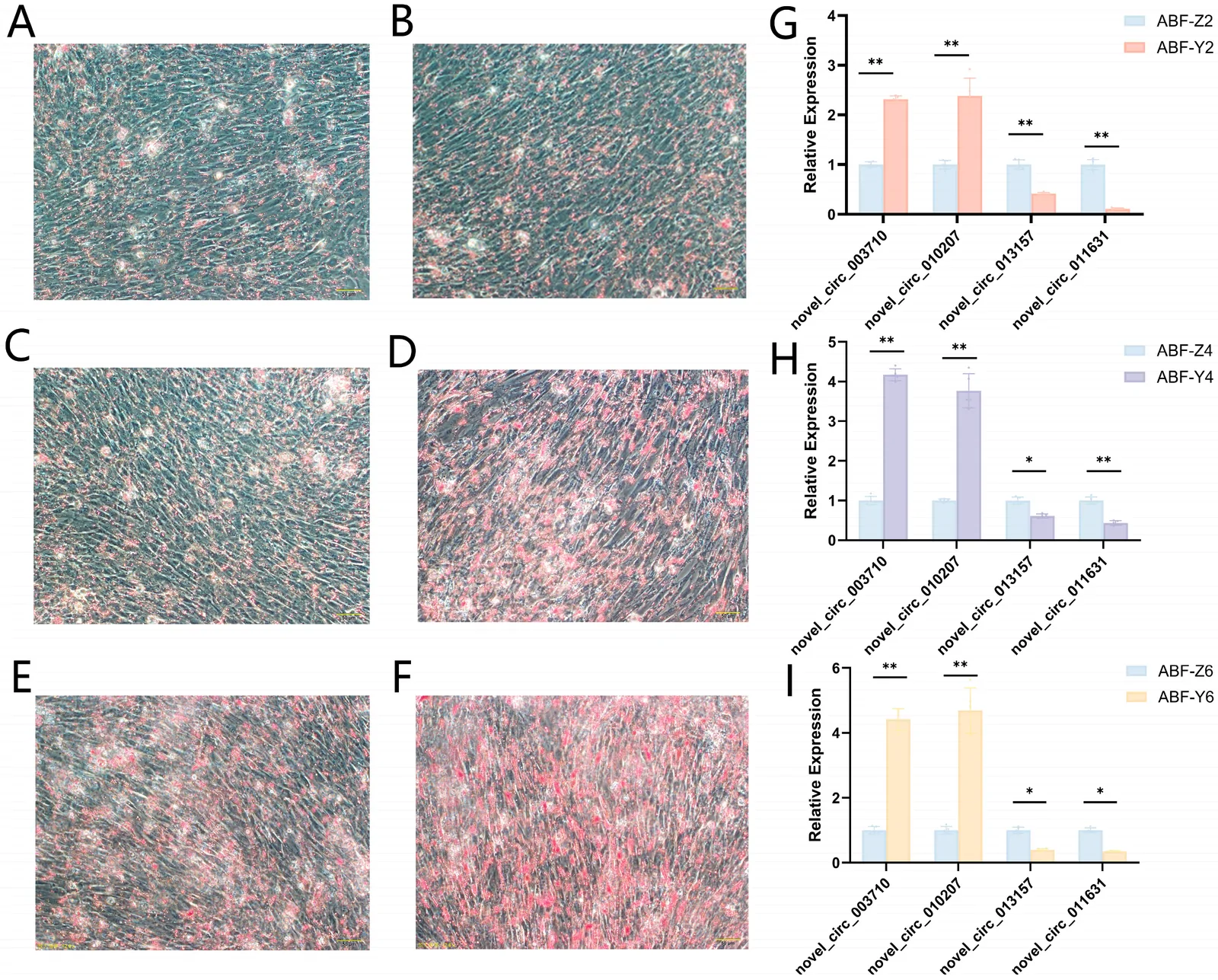
Adipogenic differentiation of primary abdominal preadipocytes and expression patterns of candidate circRNAs. (**A**–**F**) Representative images of Oil Red O-stained primary abdominal preadipocytes cultured under control or adipogenic induction conditions. (**A**,**C**,**E**) Uninduced control cells cultured for 2, 4, and 6 days, respectively. (**B**,**D**,**F**) Adipogenesis-induced cells cultured for 2, 4, and 6 days, respectively. Images were acquired using a 20× objective. Scale bar = 50 μm. (**G**–**I**) Relative expression levels of novel_circ_003710, novel_circ_010207, novel_circ_013157, and novel_circ_011631 in adipogenesis-induced cells and their time-matched controls on days 2 (**G**), 4 (**H**), and 6 (**I**), as determined by RT-qPCR. ABF-Y2, ABF-Y4, and ABF-Y6 represent cells subjected to adipogenic induction for 2, 4, and 6 days, respectively; ABF-Z2, ABF-Z4, and ABF-Z6 represent the corresponding uninduced controls. Expression in each time-matched control group was normalized to 1. Data are presented as the mean ± SEM from five independent biological replicates (*n* = 5 per group). * *p*-value < 0.05 and ** *p*-value < 0.01.

To determine whether these four candidate circRNAs are associated with adipogenesis, primary abdominal preadipocytes isolated from meat ducks were subjected to adipogenesis induction for 2, 4, and 6 days, respectively. Oil Red O staining revealed that intracellular lipid accumulation gradually increased as the induction period prolonged (Figure 6A–F). On day 2, only a small number of lipid droplets were observed in the induced cells (Figure 6A,B). After 4 days of induction, lipid accumulation became more pronounced (Figure 6C,D), and by day 6, a large number of Oil Red O-positive lipid droplets were observed in the induced cells (Figure 6E,F). In contrast, lipid staining was significantly less pronounced in the corresponding uninduced control cells. These morphological changes confirm the progression of adipogenesis in the induced primary abdominal preadipocytes.

RT-qPCR analysis revealed consistent differences in the expression of these four candidate circular RNAs between the adipogenesis-induced group and the time-matched control group (Figure 6G–I). Novel_circ_003710 and novel_circ_010207 were significantly upregulated in induced cells (*p*-value < 0.05), whereas novel_circ_013157 and novel_circ_011631 were significantly downregulated (*p*-value < 0.05). Notably, the expression patterns observed during the differentiation of primary preadipocytes were consistent with those detected in abdominal adipose tissue: novel_circ_003710 and novel_circ_010207 exhibited higher expression levels in both the HF group and adipogenesis-induced cells, whereas novel_circ_013157 and novel_circ_011631 showed lower expression levels under both conditions. These consistent expression patterns across both tissue and cellular models support an association between these four candidate circular RNAs and abdominal adipogenesis, making them priority targets for future functional studies.

### 3.9. Temporal Expression Dynamics of Candidate circRNAs During Adipogenic Differentiation

**Figure 7:**
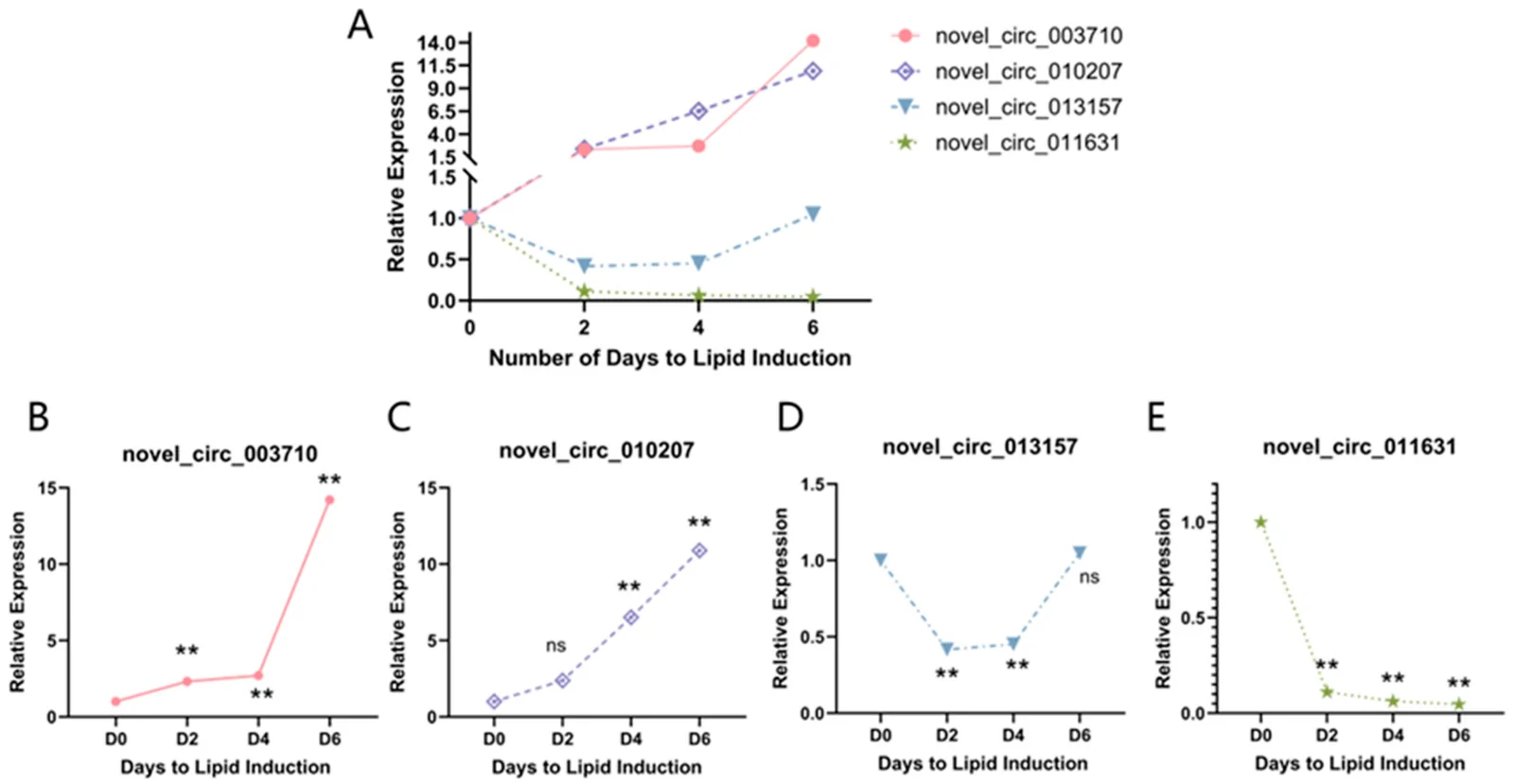
Temporal expression dynamics of candidate DE circRNAs during the adipogenic differentiation of primary duck abdominal preadipocytes. (**A**) Overall temporal expression profiles of novel_circ_003710, novel_circ_010207, novel_circ_013157, and novel_circ_011631 before adipogenic induction (D0) and after 2, 4, and 6 days of induction (D2, D4, and D6, respectively). (**B**–**E**) Individual expression profiles of novel_circ_003710 (**B**), novel_circ_010207 (**C**), novel_circ_013157 (**D**), and novel_circ_011631 (**E**), as determined by RT-qPCR. Relative expression levels were normalized to the corresponding expression levels at D0. Data are presented as the mean ± SEM from five independent biological replicates (*n* = 5 per group, Mean ± S.E.M). Statistical differences between each post-induction time point and D0 were assessed using one-way ANOVA followed by Dunnett’s test. ** *p*-value < 0.01 and ns, not significant.

To further characterize the temporal expression patterns of these four candidate circRNAs during adipogenesis, their expression levels were measured in primary abdominal preadipocytes before induction (D0) and on days 2, 4, and 6 after adipogenesis induction (corresponding to D2, D4, and D6, respectively). These four circRNAs exhibited distinctly different temporal expression profiles during differentiation (Figure 7A). The expression level of novel_circ_003710 rose significantly starting at D2 (*p* < 0.01), followed by a gradual increase at D4, and a significant rise at D6, when its expression reached the highest level observed during differentiation (Figure 7B). In contrast, novel_circ_010207 showed no significant change at D2 but was significantly upregulated at D4 and further increased at D6 (*p* < 0.01) (Figure 7C). Thus, novel_circ_003710 exhibited an early and sustained upward trend, whereas novel_circ_010207 displayed a delayed induction pattern during adipogenic differentiation. novel_circ_013157 and novel_circ_011631 exhibited different temporal patterns. Expression of novel_circ_013157 decreased significantly on D2 and D4 (*p* < 0.01), but recovered to levels comparable to those on D0 by D6 (*p* > 0.05; Figure 7D), indicating a transient decline during the early and mid-stages of differentiation. In contrast, novel_circ_011631 was sharply downregulated by D2 and remained at extremely low levels on D4 and D6 (*p* < 0.01; Figure 7E), suggesting rapid and sustained suppression throughout the differentiation process.

In summary, novel_circ_003710 and novel_circ_010207 exhibited progressive upregulation during adipogenesis, particularly during the middle and late stages; novel_circ_013157 showed a transient downregulation in the early stage, while novel_circ_011631 exhibited sustained downregulation. These distinctly different temporal expression patterns further support the association between these four candidate circular RNAs and the different stages of duck adipocyte differentiation.

## 4. Discussion

Abdominal fat is the primary site of body fat deposition and has a very high capacity for triglyceride (TG) accumulation [20,21]; its fatty acid composition is similar to that of subcutaneous fat, being rich in saturated fatty acids (SFAs) and monounsaturated fatty acids (MUFAs) [22]. Adipogenesis is governed by multilevel regulatory mechanisms encompassing both transcriptional and post-transcriptional levels [23,24,25]. In ducks, abdominal fat deposition is regulated by a coordinated, multilevel network involving fatty acid metabolism, triglyceride homeostasis, PPAR and insulin signaling, as well as the proliferation and differentiation of preadipocytes [26,27].

This study characterized the circRNA landscape of abdominal adipose tissue in two Peking duck lines divergently selected for fat deposition. The circRNAs identified in this study showed substantial heterogeneity in length and expression abundance. Most were 300–500 bp in length, and the right-skewed distribution of back-splice junction reads indicated that most circRNAs were expressed at relatively low levels, whereas only a small proportion showed high abundance. Among the 16,513 identified circRNAs, 212 circRNAs were differentially expressed between the HF group and the LF group. The equal number of upregulated and downregulated DE circRNAs further suggests that the differences in abdominal fat deposition are related to bidirectional remodeling of the circRNA transcriptome, rather than an overall increase or decrease in circRNA production. Functional enrichment analysis of DE circRNA host genes identified processes directly related to lipid storage and mobilization, including lipid-droplet organization, regulation of lipid storage, and lipoprotein and triglyceride lipase activities. Glycerolipid metabolism was among the most prominent enriched pathways, together with the regulation of lipolysis in adipocytes and pathways associated with fatty acid, sphingolipid, ether lipid, and glycerophospholipid metabolism. These processes collectively encompass lipid synthesis, remodeling, storage, and degradation and are therefore biologically consistent with the divergent fat-deposition characteristics of the HF and LF groups. AMPK, Wnt, insulin, and MAPK signaling pathways were also nominally enriched at *p*-value < 0.05. These pathways connect nutrient sensing, preadipocyte differentiation, and lipid metabolism.

Previous studies indicate that circRNA-mediated regulation of adipogenesis is partly conserved across avian species. In ducks, circ-PLXNA1 has been reported to participate in adipocyte differentiation through the miR-214/*CTNNB1* axis, providing direct evidence that circRNAs can contribute to duck adipogenesis [12]. Transcriptomic comparisons of ducks with divergent abdominal fat deposition have also identified circRNA host genes associated with PPAR signaling, lipid-droplet organization, and triglyceride metabolism [28]. In chickens, circRNA profiling during abdominal preadipocyte differentiation has revealed dynamic expression patterns and enrichment of the PPAR, fatty acid metabolism, Hippo, mTOR, insulin, FoxO, and Wnt signaling pathways [29]. Moreover, the circDOCK7/gga-miR-301b-3p/*ACSL1* axis has been functionally implicated in chicken adipocyte lipid metabolism [26]. The glycerolipid, fatty acid, insulin, Wnt, and AMPK-related signals identified in the present study therefore show pathway-level agreement with previous avian studies. However, novel_circ_003710, novel_circ_010207, novel_circ_013157, and novel_circ_011631 have not, to our knowledge, previously been reported as fat-related circRNAs in ducks or chickens. Their distinct expression patterns may represent duck-, genetic-line-, developmental-stage-, or adipose-depot-associated regulation. Nevertheless, definitive species-specific differences between ducks and galliform birds cannot be inferred solely from cross-study comparisons because circRNA detection is also influenced by breed, age, adipose depot, sequencing depth, reference-genome annotation, and bioinformatic pipelines.

The expression analysis of seven candidate DE circRNAs and their host genes revealed both concordant and discordant differential expression patterns. Novel_circ_015552/*PLPP1* were both downregulated in the HF group, whereas novel_circ_011959/*GALC* and novel_circ_016319/*HSD17B4* were upregulated. Concordant expression may reflect changes in the transcriptional activity of the host loci and the coordinated production of circular and linear transcripts from the same precursor RNA. In addition, previous studies have demonstrated that some circular intronic RNAs and exon–intron circRNAs can enhance host-gene transcription through interactions with RNA polymerase II or U1 snRNP [30,31]. However, whether such mechanisms apply to the circRNAs identified here depends on their genomic origin, subcellular localization, and molecular interactions and cannot be determined from expression direction alone. *PLPP1* participates in lipid phosphate metabolism and associated signaling, *GALC* contributes to lysosomal sphingolipid degradation, and *HSD17B4* encodes a multifunctional enzyme involved in peroxisomal fatty acid β-oxidation [32,33,34]. Thus, the concordant changes in these circRNA–host-gene pairs may reflect coordinated regulation of lipid signaling, sphingolipid turnover, and fatty acid oxidation in abdominal adipose tissue.

Four other circRNA–host-gene pairs showed discordant expression. Novel_circ_005155 and novel_circ_012415 were upregulated in the HF group, whereas their corresponding host transcripts, *LOC101802708* and *PNPLA2*, were downregulated. Conversely, novel_circ_004386 and novel_circ_005696 were downregulated, whereas *CFTR* and *ALDH3A2* were upregulated. The production of circRNAs through back-splicing can compete with canonical pre-mRNA splicing under some conditions, potentially resulting in opposing changes in circular and linear transcript abundance [35,36]. Differences in RNA stability, degradation, transcription rate, and cell-type composition may also contribute to discordant expression. Of particular interest, *PNPLA2*, also known as adipose triglyceride lipase, catalyzes the initial step of intracellular triglyceride hydrolysis [37]. The increased abundance of novel_circ_012415 together with reduced *PNPLA2* expression in the HF group is therefore consistent with, but does not demonstrate, a possible shift between circular and linear transcript production at this locus. Reduced *PNPLA2* expression may be associated with decreased triglyceride mobilization and increased lipid retention; however, actual lipolytic activity cannot be inferred from mRNA abundance alone. *ALDH3A2* is involved in the oxidation of fatty aldehydes generated during lipid metabolism [38], whereas *LOC101802708* is annotated as a putative PNPLA-like locus and should not be considered equivalent to *PNPLA2*. Overall, the coexistence of concordant and discordant expression patterns indicates that circRNA abundance is influenced by transcription, alternative splicing, back-splicing, and RNA stability.

Fat deposition depends on the proliferation, differentiation, and maturation of preadipocytes, and preadipocytes derived from the abdomen exhibit a high potential for adipogenic differentiation [39]. The four circRNAs selected from the predicted ceRNA network represent a complementary layer of candidate prioritization rather than direct extensions of the seven circRNA–host-gene pairs described above. Primary abdominal preadipocyte experiments provided additional evidence linking them to adipocyte differentiation. Oil Red O staining revealed a gradual increase in lipid droplet accumulation 2, 4, and 6 days after induction. At all time points, the expression levels of novel_circ_003710 and novel_circ_010207 were higher in induced cells than in the control group, whereas the expression levels of novel_circ_013157 and novel_circ_011631 were lower. Importantly, these trends were consistent with the tissue comparison results: novel_circ_003710 and novel_circ_010207 were upregulated in both abdominal adipose tissue and differentiating adipocytes in the HF group, whereas novel_circ_013157 and novel_circ_011631 were downregulated under both conditions. This consistency across models reinforces their association with abdominal adipogenesis and reduces the likelihood that tissue differences are due solely to variations in cell composition.

Temporal analysis further distinguished these four candidate genes. Novel_circ_003710 began to rise starting on D2 and peaked on D6, indicating an early and sustained response to adipogenic differentiation, followed by a significant increase during the late stages of differentiation. The level of Novel_circ_010207 at D2 did not differ significantly from that at D0, but it increased at D4 and D6, indicating delayed induction during the middle and late stages of adipogenesis. In contrast, novel_circ_013157 showed a transient downregulation at D2 and D4 but had returned to baseline levels by D6, whereas novel_circ_011631 exhibited rapid and sustained suppression throughout the differentiation process. These expression patterns suggest that these four circular RNAs are associated with different stages of adipocyte differentiation rather than exhibiting a uniform response to lipid induction. Similar stage-dependent circRNA expression has been reported in chicken, cattle, sheep, and mammalian adipocyte models [26,29,40,41,42]. From a breeding perspective, the four prioritized circRNAs provide candidate molecular resources for investigating genetic variation in abdominal fat deposition, but they cannot yet be regarded as directly applicable selection markers. Measurement of circRNA expression in abdominal adipose tissue requires invasive or post-mortem sampling, which limits its direct use in live-animal selection. Future studies should first validate the associations of these circRNAs with abdominal fat traits in larger independent populations encompassing both sexes, multiple developmental stages, and additional genetic backgrounds.

Several limitations should be considered when interpreting these findings. The RNA sequencing sample size (*n* = 4 per group) was constrained, and although expanded via RT-qPCR (*n* = 8 per group), validation in a larger independent population remains necessary. Second, sex was not determined at the time of sampling, and possible sex-related variation in adipose development or circRNA expression therefore cannot be excluded. Third, HF and LF were not groups formed by selecting phenotypically extreme individuals from the current cohort. Instead, they were genetic groups established through multiple generations of bidirectional selection at the Institute of Animal Science, Chinese Academy of Agricultural Sciences. The LF group is characterized by a high lean-meat percentage and low abdominal fat, whereas the HF group shows higher body fat content and more rapid subcutaneous and abdominal fat deposition. Consequently, the observed differences should be regarded as line-associated, because the effects of abdominal fat deposition cannot be completely separated from broader differences in genetic background. Fourth, the use of 14-day-old ducks limits the conclusions to an early developmental stage of adipose tissue and does not establish whether the same circRNA patterns persist at later growth or commercial slaughter ages. Finally, primary preadipocyte differentiation in vitro does not fully reproduce the endocrine, nutritional, vascular, and cellular environments of abdominal adipose tissue in vivo. These limitations warrant cautious interpretation of the identified circRNAs as candidates rather than confirmed regulators of abdominal fat deposition.

In summary, this study mapped the circRNA profiles of abdominal adipose tissue in Peking duck HF and LF groups and identified DE circRNAs associated with lipid metabolism and fat deposition. By integrating host gene enrichment analysis, predicted ceRNA interactions, tissue expression data, and primary cell differentiation data, we found that novel_circ_003710 and novel_circ_010207 were positively correlated with adipogenesis, whereas novel_circ_013157 and novel_circ_011631 exhibited negative or stage-dependent associations. These circRNAs constitute biologically relevant candidate molecules for elucidating the post-transcriptional regulatory mechanisms of abdominal fat deposition in ducks. However, the potential application of these candidate circRNAs as molecular biomarkers or regulatory targets requires further experimental validation. The circRNA/miRNA/mRNA interactions revealed by Pearson correlation analysis in this study merely suggest potential co-expression or competitive binding trends and do not directly demonstrate bona fide ceRNA regulatory mechanisms. However, their molecular interactions, causal functions, and potential breeding applications require further experimental validation.

## 5. Conclusions

This study systematically analyzed circRNA expression profiles in the abdominal adipose tissue of Peking ducks with different abdominal fat percentages, identifying a total of 212 differentially expressed circRNAs. Their host genes were significantly enriched in signaling pathways related to glycerol and fatty acid metabolism, lipid storage and mobilization, and adipogenesis. Tissue and cellular validation revealed that novel_circ_003710 and novel_circ_010207 may be positively correlated with abdominal fat deposition and adipocyte differentiation, whereas novel_circ_013157 and novel_circ_011631 were negatively or stage-specifically correlated. These findings establish a dynamic expression landscape of candidate circRNAs in abdominal adipose tissue and primary preadipocytes, providing valuable candidate targets associated with abdominal fat deposition and early preadipocyte differentiation in Peking ducks.

## Figures and Tables

**Table 1:** Body weight, abdominal fat weight, and abdominal fat percentage for sequencing in the test groups.

Group	Live Body Weight (g)	Abdominal Fat Weight (g)	Abdominal Fat Percentage (%)
HF	571.75 ± 21.34 ^a^	3.19 ± 0.38 ^a^	0.56 + 0.05 ^a^
LF	539.5 ± 37.22 ^a^	0.59 ± 0.16 ^b^	0.11 ± 0.02 ^b^

Different lowercase letters in the same column indicate statistically significant differences (*p*-value < 0.01), while the same letter indicates no statistically significant difference (*p*-value > 0.05). Data are presented as the mean ± SEM.

## Data Availability

All sequencing data supporting the findings of this study are available in the NCBI SRA repository under BioProject PRJNA1497502.

## Supplementary Materials

The following supporting information can be downloaded at: https://www.mdpi.com/article/10.3390/biology15161405/s1. Table S1: The primers used for qRT-qPCR; Table S2: Phenotypic and sampling information for individual HF and LF Peking ducks used in circRNA sequencing and RT-qPCR validation; Table S3: Data Filtering Statistics; Table S4: Base Information Statistics: Table S5: Ribosomal Alignment Statistics; Table S6: Statistical Summary of Genome Alignments; Table S7: Comparison of Statistical Data for Reference Areas; Table S8: DE circRNAs between the HF and LF groups; Table S9: Analysis of ceRNA Regulatory Relationships.

## Abbreviations

The following abbreviations are used in this manuscript:

ABF	Abdominal fat
*PLPP1*	Phospholipid phosphatase 1
*ALDH3A2*	Aldehyde dehydrogenase 3 family member A2
*GALC*	Galactosylceramidase
*PNPLA2*	Patatin-like domain 2, triacylglycerol lipase
*CFTR*	CF transmembrane conductance regulator
*HSD17B4*	Hydroxysteroid 17-beta dehydrogenase 4
*LOC101802708*	Patatin-like phospholipase domain-containing protein 2
